# Langmuir-Based Modeling Produces Steady Two-Dimensional Simulations of Capacitive Deionization via Relaxed Adsorption-Flow Coupling

**DOI:** 10.1021/acs.langmuir.1c02806

**Published:** 2022-03-08

**Authors:** Johan Nordstrand, Joydeep Dutta

**Affiliations:** † Functional Materials Group, Applied Physics Department, School of Engineering Sciences, KTH Royal Institute of Technology, AlbaNova Universitetscentrum, 106 91 Stockholm, Sweden; ‡ Center of Nanotechnology, King Abdulaziz University, 21589 Jeddah, Saudi Arabia

## Abstract

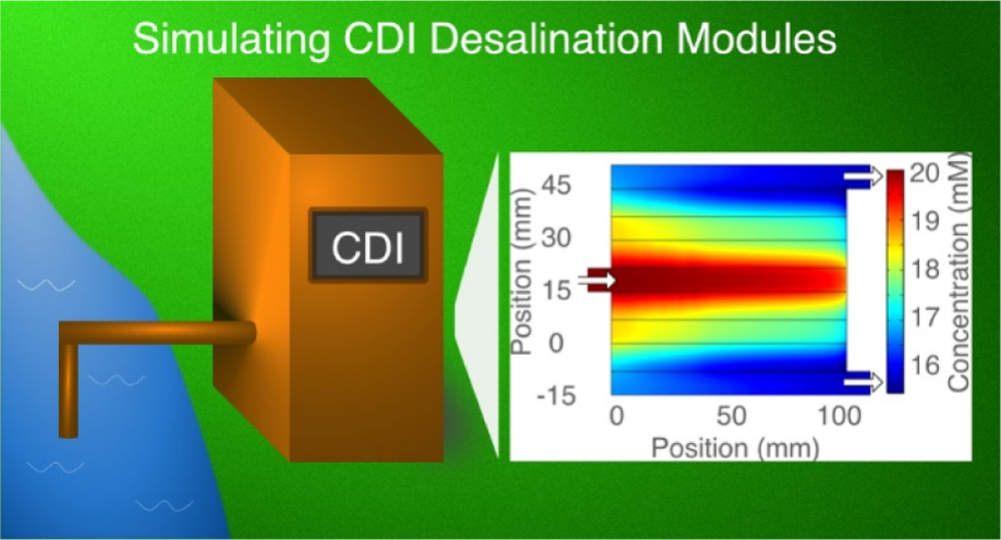

The growing world population creates an ever-increasing demand for fresh drinkable water, and many researchers have discovered the emerging capacitive deionization (CDI) technique to be highly promising for desalination. Traditional modeling of CDI has focused on charge storage in electrical double layers, but recent studies have presented a dynamic Langmuir (DL) approach as a simple and stable alternative. We here demonstrate, for the first time, that a Langmuir-based approach can simulate CDI in multiple dimensions. This provides a new perspective of different physical pictures that could be used to describe the detailed CDI processes. As CDI emerges, effective modeling of large-scale and pilot CDI modules is becoming increasingly important, but such a modeling could also be especially complex. Leveraging the stability of the DL model, we propose an alternative fundamental approach based on relaxed adsorption-flow computations that can dissolve these complexity barriers. Literature data extensively validate the findings, which show how the Langmuir-based approach can simulate and predict how key changes in operational and structural conditions affect the CDI performance. Crucially, the method is tractable for simple simulations of large-scale and structurally complex systems. Put together, this work presents new avenues for approaching the challenges in modeling CDI.

## Introduction

As the world population continues to grow, the global need for freshwater provision will steadily increase.^1^ Due to the tiny fraction (<1%) of readily available freshwater in rivers, lakes, and groundwater,^2^ it is imperative to tap into the abundantly available saline water bodies by constructing effective desalination technologies,^3−11^ such as the emerging capacitive deionization (CDI)^12−17^ technique.

**Figure 1:**
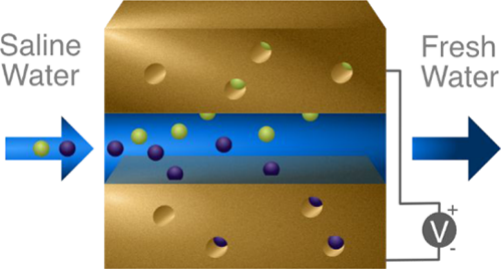
Illustration of a flow-between CDI cell.^18^ The cell comprises two porous electrodes separated by a spacer region. The applied voltage effectively pulls the ions and makes them adsorb onto the electrodes.

A CDI device comprises two porous electrodes separated by a non-conducting spacer. During desalination, an applied potential precisely extracts salt ions from the water to adsorb them inside the spacious electrodes^18^ (Figure 1). The process is strongly susceptible to changes in operational and structural conditions, such as the voltage,^19−21^ ion concentration^22−24^ and composition,^25−35^ flow rate,^36,37^ electrode material,^38−47^ electrode modifications,^40,48^ device structure,^49−54^ flow mode,^55,56^ and presence of ion-selective membranes.^16,32,57^ Also, the ion-concentration dependency makes the intracellular dynamics important.^18^ Thus, researchers have developed models that capture some of this spatial information, such as in 1D,^58−60^ partial 2D,^61^ or full 2D.^18,62^

Most modern modeling of CDI focuses on how ions are stored in electric double layers (EDLs) on an electrode surface, for example, the modified Donnan (mD)^12,13,18,22^ model. However, while proponents of such models have argued that this is the only correct approach (ref (13), Figure 12), multiple earlier works have achieved surprisingly accurate results when using a simple Langmuir isotherm to describe equilibrium adsorption.^63−69^ This raised the question of whether the results were merely a coincidence, a numerical effect, or if the Langmuir model has physical relevance for the CDI system. Investigating this, we recently introduced a dynamic Langmuir (DL) model.^24^ The work presented the surprising results that Langmuir-based modeling could generate both accurate and physically meaningful results, at equilibrium^24,30,33^ and in the 0D setting.^23,70^

This work will build on those results by investigating if a Langmuir-based approach can simulate detailed microscopic results or if the approach is only valid on the macroscale. Thus, we derive and implement a DL model in 2D. While earlier authors on 2D modeling have noted that such models can be “unsteady”,^18^ the early DL approach had a decoupled framework that enhanced the stability in lower dimensions.^23,24,70^ Thus, we develop two decoupled approaches that could enhance the stability of spatial CDI modeling to demonstrate the underlying stabilizing principles. The first is based on an older circuit-modeling framework, while the other uses the new DL model. Crucially, the work emphasizes the extra stability that the Langmuir-based modeling can bring and its implications for the modeling of upscaled devices.

## Theory

Complex and heavily coupled systems can be computationally difficult to solve. Therefore, the theory section will present equations that are required to implement the two decoupled modeling approaches. The first is based on circuit modeling, while the second uses the DL model.

### Randles Circuit

**Figure 2:**
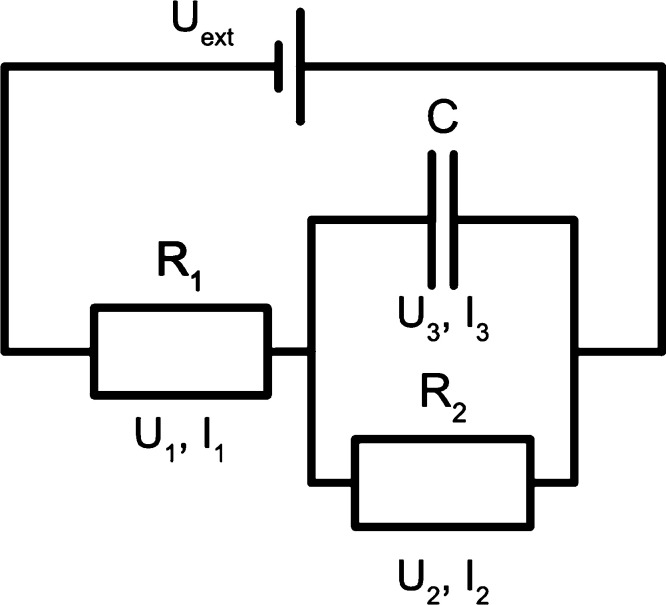
Schematic showing the Randles-circuit representation of a CDI system. The capacitance *C* determines the charge storage capacity, the parallel resistance *R*_2_ represents leakages, and the series resistance *R*_1_ represents all effects limiting the charging rate.

As we begin to describe CDI modeling, a simple model that comes to mind is an electrical circuit (Figure 2).^71^

In the Randles circuit, the capacitive element *C* firmly determines the maximum charge storage capacity, while the resistive component *R*_2_ conversely determines the charge that uselessly leaks past the electrodes. This leaves the *R*_1_ series resistance, which collectively describes the ion diffusion, electrode kinetics, and solution resistance. The importance of this picture is that it will allow for a compact description of the CDI charging in 0D.

The equations below show the specific charging dynamics depending on the charging state and the applied voltage (eqs 1–3).
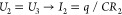
1
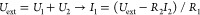
2
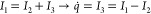
3

In these equations, *q* is the charge on the capacitor and *U*_ext_ is the total potential. Also, *U*_*i*_, *I*_*i*_, and *R*_*i*_ are the potential, current, and resistance across the components *i* = {1, 2, 3} as shown in Figure 2. Equation 1 stems from the parallel connection, eq 2 is related to the serial connection, and eq 3 represents the conservation of charge. The point here is that the Randles circuit can simulate charging as a self-contained unit. Hence, coupling it with a description of the charge–adsorption relationship would make it possible to describe ion adsorption on the electrodes.^72^

### Dynamic Langmuir Model

An alternative approach to the Randles circuit is the DL model. This work will demonstrate the performance of both within the relaxed-coupling framework.

The original Langmuir-adsorption model describes the adsorption rate of gas molecules on plain surfaces (eq 4).^73^ The equilibrium version of this expression is the classic Langmuir isotherm (eq 5).
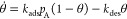
4
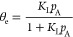
5

In these equations, θ is the fractional surface coverage, *k*_ads_ and *k*_des_ are constants, (1 – θ) corresponds to the fraction of free sites, *p*_A_ is the partial pressure, *K*_L_ = *k*_ads_/*k*_des_ is a constant, and subscript e denotes the corresponding equilibrium quantity. The insight here is that we can view adsorption processes as a balance between adsorption and desorption strengths. Also, the total adsorption can be limited by the total effective number of surface sites. These ideas will be important in the following sections.

Here, substituting *p*_A_ for the concentration *c* allows the isotherm to incorporate ion adsorption in liquids as well.^74^ Thus, researchers have subsequently noted that it comfortably describes the concentration dependence of electroadsorption of ions in CDI.^63−69^ Because it works for describing ionic concentrations during CDI, later work successfully altered and extended the classical Langmuir isotherm into a DL model.^23,24,30^ This means that the DL model can widely and precisely predict CDI performances over a broad range of operational and structural conditions.^23,24,30^

The DL model starts from a similar idea to the classic Langmuir adsorption but assumes that the core mechanisms have shifted from passive gas adsorption to electrically mediated adsorption of charged species. Here, eq 6 thus shows the adsorption rate for charged species. The corresponding modified expression for net adsorption is shown in eq 7.
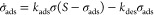
6
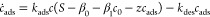
7

In the equations above, *c* is the concentration, σ is the concentration of charged species (σ = *zc*, *z* is the valency), *S* is the effective number of sites, and subscript ads denotes a corresponding adsorbed quantity. Also, β_0_ and β_1_ are constants, and *c*_0_ is the initial concentration. The interpretation here is that all charges are driven to absorb by the voltage, but only the net adsorption is valuable for desalination. A variety of effects can cause non-ideal charge efficiency.^55^ Because these effects have a varying impact, we consider coion expulsion as the major effect, and coions can be present either to neutralize the fixed charge on the electrodes^25^ (the term with β_0_) or passively through the solution concentration (the term with β_1_*c*_0_). These effects make the applied voltage expel coions rather than adsorbing the counterions, which reduces the effective number of sites for adsorption.

Here, it is worthwhile to stop and consider the physical picture. Previous models in CDI commonly consider adsorption onto EDLs in the tiny micropores. For instance, the mD model notes that the pore sizes are so small compared to the ions that the EDL heavily overlaps (see the picture in ref (22)), so the main contribution to the capacitance is the tightly packed Stern layer.^18^ In analogy, the DL model describes the same adsorption but in terms of the effective number of electroadsorption sites that the Stern layer corresponds to.

Based on the expression above, the isotherm expression looks somewhat different in CDI. The expression for the charge storage has the classic Langmuir trend but the expression for the net adsorption must include the losses.
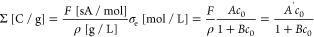
8
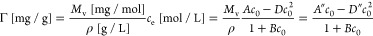
9
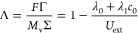
10

In the equations above, Σ is the specific charge storage, Γ is the specific adsorption, and Λ is the charge efficiency. The conversion parameters listed are the Faraday constant *F*, the electrode density ρ, and the molar weight *M*_v_. Also, the model parameters *A*, *B*, *D*, λ_0_, and λ_1_ (including the variations with ′ and ″) are constants. The reason the external voltage appears in the expressions above is that the sites *S* are proportional to the applied voltage. Note that the voltage also affects the *A* parameters through the same relationship (see eqs 7 and 8). The point here is that the expression provides a simple and decoupled method of describing charge and adsorption. For instance, the adsorption is negligible if *S* < (β_0_ + β_1_*c*_0_). Thus, when solving for the equilibrium state, the specific charge storage is proportional to the applied voltage, while the specific ion adsorption varies linearly with the applied voltage. Despite the various parameters presented to make the expression decoupled, note that the entire system has only three degrees of freedom.

Previous 2D-coupled CDI research has profitably examined diluted ionic concentration (low *c*) conditions and shown that 2D spatially resolved modeling can be important to examine in this regime. Therefore, it is interesting to look at what the DL model would correspond to under such conditions (eqs 11–13).
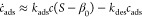
11
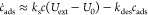
12
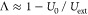
13

The new parameters in these expressions are the constant *k*_s_ = *k*_ads_*S* and the baseline voltage *U*_0_, which are system-dependent parameters. The derivation follows from the full expression by setting the initial concentration to zero. For comparison, low pressure *p*_A_ in the Langmuir isotherm means *K*_L_*p*_A_ ≪ 1, making the adsorption increase proportionally to the pressure. Similarly, the same effect can be seen in the DL model (see Figure 2 in ref (24)). Here, low concentration means *k*_ads_*cz* ≪ *k*_des_ and β_1_*c*_0_ ≪ β_0_.

Earlier work has demonstrated that a system-identification method can directly extract all model parameters based on dynamics extracted from the experimental data.^23,70,71^ However, having equilibrium data could simplify the extraction process. Let us consider experimentally determined conditions as *c*_ads,cal_ (experimental calibration adsorption) and Λ_cal_ at *U*_cal_. Then, eq 13 makes *U*_0_ = *U*_cal_(1 – Λ_cal_) and, because *ċ*_ads_ = 0 at equilibrium, *k*_des_ = *k*_s_*c*_0_(*U*_cal_ – *U*_0_)/*c*_ads,cal_. Thus, *k*_s_ is the system’s only remaining unknown parameter, which COMSOL’s optimization module confidently extracts using the entire time-series data.

### Flow Modeling

The previous sections showed two separate modeling approaches for systematically deducing the local adsorption rate in a typical CDI device. The next step is to couple an adsorption model to a flow-dependent ion-transport model in order to mimic the real-life situation.

Let us, therefore, begin to focus on ion transport. The core principle of transport is the mass transport equation, which states that the change in concentration depends on the difference between the concentration flow in and out of a region. In a well-stirred container, the same principle holds for a bulk volume, which leads to a 0D description of the flow.
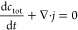
14
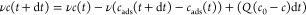
15
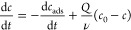
16Here, *j* is the ion flux, *Q* is the flow rate, the total concentration is denoted *c*_tot_ = *c* + *c*_ads_, and ν is the free volume for the water in the device. The idea here is that it is possible to reduce a fully spatial model to lower dimensions by assuming that the concentration is approximately uniform across these dimensions. Hence, the impact of the flow rate depends on how quickly the total volume in the device is exchanged with new water.

An interesting feature of the modeling approach is that it could be implemented in any dimensionality. The adsorption equations (such as eq 12) are completely local, so there is no strict requirement that the model must include certain spatial information. Rather, the spatial dimension that is included allows the model to describe changes in adsorption rate and concentration along that dimension. Thus, a 0D assumes equal adsorption or concentration everywhere. A 1D model assumes equal adsorption and concentration throughout the depth and either length or thickness of the device. Finally, a 2D model would assume homogeneity throughout the depth and a 3D model would include all spatial dimensions. In this work, we will focus on 2D modeling.

## Methods

### Implementation

As this work’s primary goal is to effectively model 2D systems through a relaxed adsorption-flow coupling, two separate approaches will be provided.

In the first approach, a decoupled method superimposes separately solved adsorption and flow problems. While this may initially seem like a naive approach, the findings in the result section made us realize that it yields surprisingly accurate computations while, crucially, achieving the computational stability required for large-scale modeling. This finding arises largely because the separate adsorption calculation still contains components representing size-dependent effects, such as *R*_1_ representing diffusion-limited adsorption rates (Figure 2).

In practice, the Randles circuit could be used to simulate adsorption by fitting its parameters to cell-dependent current data in combination with measuring the charge efficiency. Alternatively, a 0D adsorption-flow approach can fit the Randles circuit to combined the current and adsorption data. Because the model is now fitted, it can predict the 0D time-varying adsorption and simulate the effects of varying operational or structural conditions. Subsequently, having the adsorption prediction means a full-2D COMSOL flow simulation can uniformly use it as an input reaction rate to reliably simulate the relaxed coupled process.

The second approach holistically simulates adsorption and flow together and loosely couples them by softly relaxing the computational complexity. This relaxation is achieved in two ways by practically integrating the DL electroadsorption model (eq 12) into the COMSOL’s reaction module. First, the DL model locally determines the adsorption using ordinary differential equations (ODE), in contrast to previous models that compute potential and ion-concentration distributions in extensive connected partial differential equations (PDE).^18,62^ The “relaxed” part of relaxed coupling here means that the model is fully 2D while using less spatial information, enhancing computational stability. Second, we simplifyingly assume that the adsorption–desorption constants are uniform throughout the electrode volume, which limits the system’s degrees of freedom. This also lets system-identification methods automatically extract the model parameters.

### Simulations

**Figure 3:**
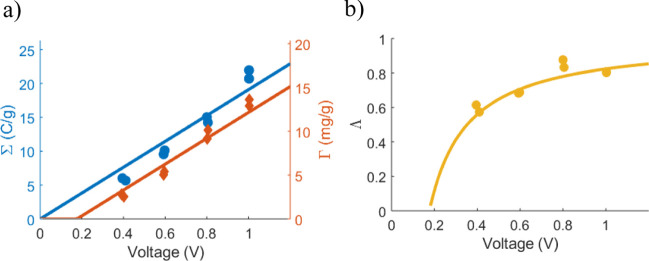
Equilibrium performance of a flow-between CDI cell, with experimental data from ref (18). Model lines show the fit to data, assuming the relationship between capacitance and adsorption as in eqs 8 and 9. (a) Specific charge storage and salt adsorption. (b) Charge efficiency for the charge and adsorption in (a) (eq 13).

**Figure 4:**
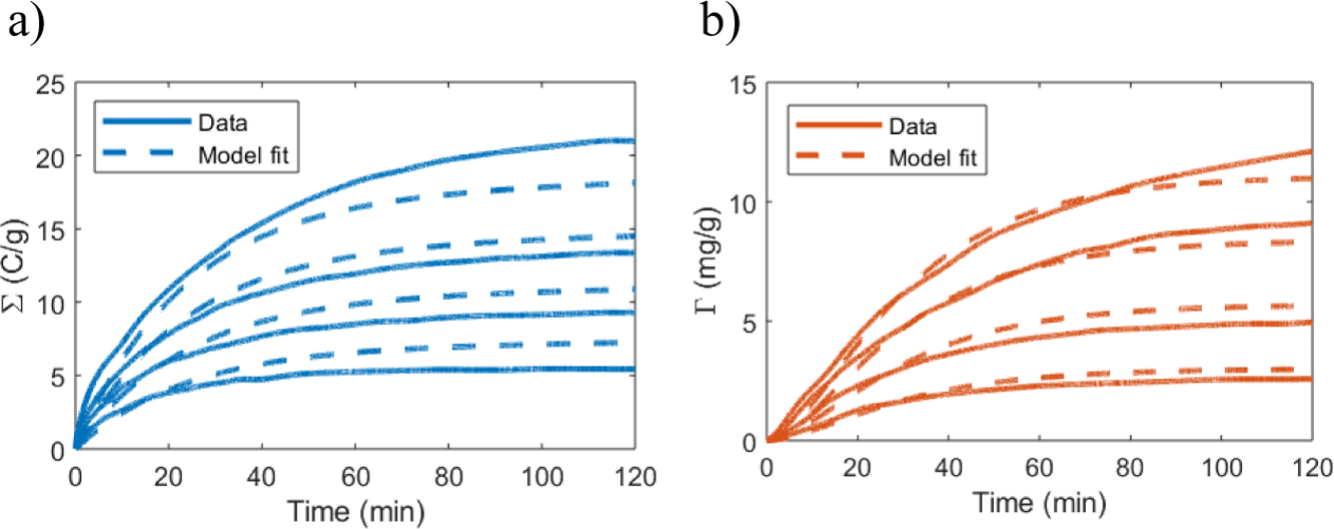
Using the known capacitance and charge efficiency from Figure 3, a system-identification method can fit the rest of the model parameters to voltage-dependent time-varying performance. Here, the Randles model was fitted to data for all voltages simultaneously. All experimental data from ref (18). (a) Time-varying charge storage. (b) Time-varying total adsorption.

In this work, the 0D computation was implemented in MATLAB (Figures 3 and 4). Within MATLAB, the system-identification toolbox implements a non-linear gray-box estimation (nlgreyest), which allows the user to automatically find the model parameters that yield the best fit to one or more sets of time-series data. For this, it requires a model formulation and fitting data. Here, we use the Randles circuit (Figure 2) with the formulation from eqs 1–3.

For the coupling, the implementation in COMSOL handles all flow simulations and adsorption-flow couplings using the standard framework for “Reacting Flow in Porous Media”. Thus, the Brinkman equations solve the free- and porous-media flow inside the cell (“Brinkman Equations”), while mass transport equations determine the ionic movement (“Transport of Diluted Species in Porous Media”).

The “Reactions” interface under “Transport of Diluted Species in Porous Media” calculates salt adsorption by treating it as a reaction in which the free ion concentration was converted to the adsorbed ion concentration. The decoupled model uses an “Interpolation Function” to generate a time-dependent adsorption rate based on previously externally calculated adsorption rates. The loosely coupled DL-based model instead uses ODEs and a data envelopment analysis “Domain ODE and DEA” module to implement a distributed differential formulation (eq 12) to track the total adsorption separately from the free concentration. In the latter case, the “Optimization” module automatically generates the best adsorption rate constant based on the uploaded reference data.

### Validation Data

The result section extensively examines and validates the model using data from reports in the literature. Among these reports, we primarily considered the seminal work by Hemmatifar et al. because it is the major report on full 2D coupling for CDI using finite-element simulations.^18^

## Results and Discussion

Full 2D modeling often uses extensive sets of coupled PDEs to simulate the device performance.^18,62^ However, the complexities^18^ can limit the possible parameter-extraction methods and poor convergence can cause programs to crash. This lack of stability constrains the model’s practical usefulness for feasibly predicting the performance, especially of large-scale and complex CDI structures. In contrast, this section will procedurally demonstrate how partially or fully decoupling the adsorption and flow dramatically enhances stability while maintaining effective modeling accuracy.

### Flow-Independent Modeling

#### Equilibrium Charge and Adsorption

This section seeks to validate the core model before moving to coupled simulations. For the equilibrium case, the DL model predicts that the stored charge increases proportionally with the voltage through the voltage-induced sites. Similarly, the adsorption increases linearly with the voltage but the electrode must overcome a blockage before being able to achieve net adsorption. The results show that the equilibrium DL model accurately fits the equilibrium charge (eq 8, Figure 3a), adsorption (eq 9, Figure 3b), and charge efficiency (eq 10, Figure 3b), without introducing any notion of water flow or ionic transport.

Specifically, the average error magnitude is 1.8 C/g or 1.1 mg/g. The absolute error is fairly constant, so the relative error decreases for higher voltages (e.g., 25 to 8% for the adsorption). The average relative error magnitude is 17.2% for the charge and 14.5% for the adsorption.

Compared to the previous study with the mD model,^18^ the performance of the DL model is slightly better for describing the experimental results. On the one hand, the mD model includes more details that allow it to describe the performance below 0.4 V in more detail. On the other hand, the linear trends in the DL model are less sensitive to the non-constant capacitive effects at higher voltages, leading to an overall better agreement with data.

#### Time-Varying Charge and Adsorption

This section continues from the last section by showing that the Randles circuit (in addition to the DL model) can simulate time-dependent performance. The core Randles circuit represents the time-varying adsorption through a resistive element (Figure 2). Thus, flow-dependent effects, such as slow diffusion limiting the adsorption rate, are represented as increased resistance.

Using the equilibrium data in Figure 3 to determine the capacitance and charge efficiency, the Randles circuit directly fits the resistance parameters to accurately capture these experimental time variations (Figure 4). There are some discrepancies between the model and experimental performance, which correspond directly to the non-linear capacitance in the equilibrium data. However, the key point to notice is that the time-dependent trends are accurately captured without introducing detailed coupled flow-adsorption calculations.

Quantitatively, the error in adsorption at equilibrium (120 min) is 13.7% at 0.4 V, 12.2% at 0.6 V, 9.4% at 0.8 V, and 10.3% at 1.0 V. These error rates are stable at around 10% after *t* = 20 min. The corresponding values for the charge are 25.2% at 0.4 V, 14.6% at 0.6 V, 7.9% at 0.8 V, and 15.8% at 1.0 V. These errors are also somewhat stable during the process.

Compared to previous mD modeling results,^18^ the modeling here shows better agreement with data. A minor effect comes from the better agreement at the equilibrium level (Figure 3). However, the main reason is that the circuit model is better at describing the overall time scales of charging and adsorption, whereas the mD model predicted too slow charging for the same data set. Hence, the Randles circuit managed well to describe the overall rate-limiting factors (the circuit resistance), and the system-identification process produced great values for the model parameters.

### Flow Modeling

**Figure 5:**
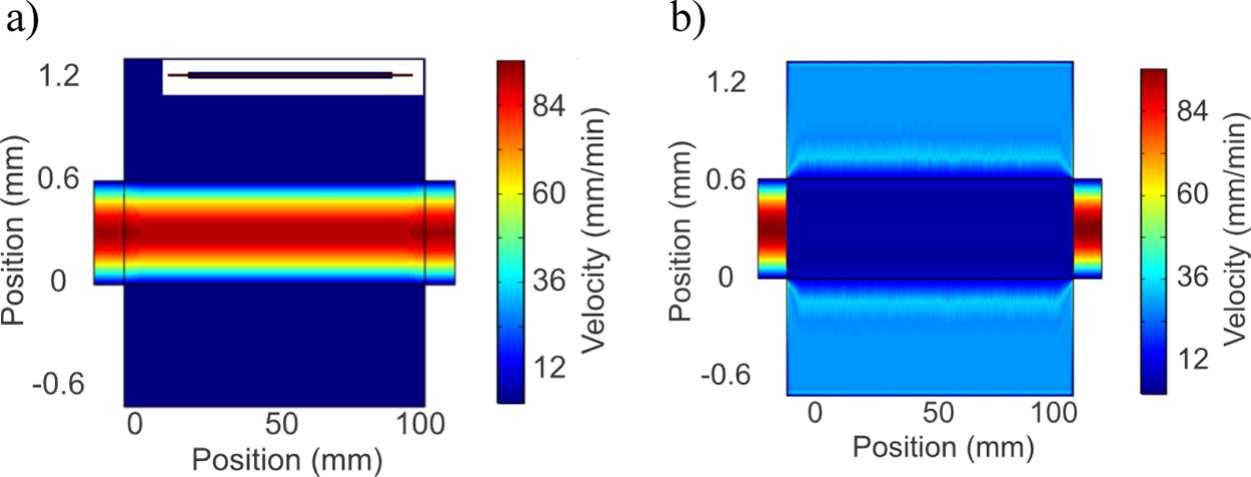
(a) Image shows simulated flow based on the cell structure and operational conditions in ref (18). (a, inset) To give a sense of proportion, the inset shows the flow-between cell in (a) with equal *x-* and *y*-axis scaling. (b) Simulated flow corresponding to the cellulose spacer in ref (49) being used instead of an air gap as in ref (18). The results suggest that the relative permeability between the spacer and the electrode is crucial for determining the flow path.^18^

FEM software has the advantage of being able to simulate the flow inside the device even if the device structure is complex. In CDI, the relative permeability of the spacer and electrode will have a significant impact on the flow pathway. Figure 5a shows that all flow goes through the spacer regions if that region is just an air gap. This agrees with previous findings.^18^Figure 5b instead shows that most of the flow passes the electrode if the spacer is less permeable than the electrodes. This is the expected result based on the permeabilities, and a further discussion of similar flow pathways is presented in ref (53).

### Coupled Modeling

#### Adsorption Source in 0D

The previous sections demonstrate that it is possible to simulate flow and adsorption separately with reasonable results. However, the adsorption model did not contain any spatial information about what is actually going on in different parts of the device. To add this feature, the simplest way of coupling them to start investigating spatial adsorption/transport processes would be to superimpose the adsorption on the flow simulation.

**Figure 6:**
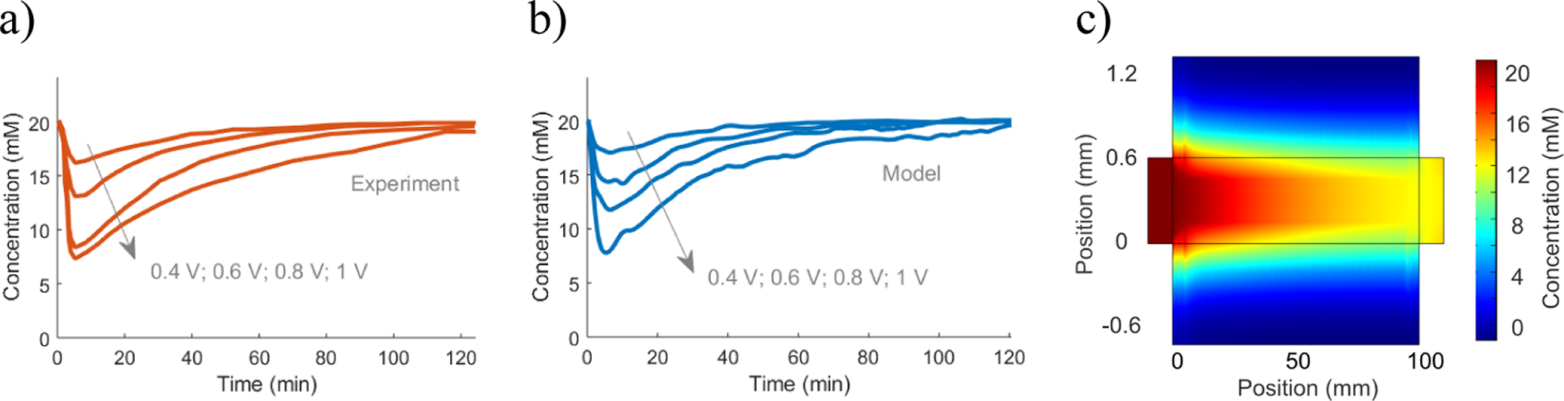
(a) Effluent ion concentration for a flow-between CDI cell, experimental data from ref (18). (b) Knowing the total time-dependent adsorption from Figure 4 lets us use this adsorption as an input source in a flow model to predict the effluent ion concentration. (c) For highest voltage (1 V),^18^ the decoupled model suggests that a uniformly distributed adsorption would abruptly cause complete local depletion after only 2 min, which means the low concentration would severely limit the real system’s effective adsorption rate.

That is, knowing the total adsorption rate (Figure 4) and the flow distribution (Figure 5), one method of weak coupling is to assume a uniform adsorption distribution across the electrode. This simplified approach neglects detailed localized variations but, because the correct total current and adsorption are used as the input, the model accurately still captures the global effluent-concentration trends (Figure 6a,b). Instead, the neglected detailed variation may cause the model to continue uniformly removing ions even if the adsorption should locally slow down because the concentration is depleted at that location. While this might initially seem like an error, notice that a locally unreasonably low concentration becomes a clear indicator of a concentration shock (Figure 6c). This means the weakly coupled model fulfills the stated goal of identifying concentration shocks while yielding good detailed results for non-starved conditions and being globally accurate.

The relative error across all voltages is 5.9%. On a detailed view, the errors are 2.1% at 0.4 V, 2.4% at 0.6 V, 9.4% at 0.8 V, and 8.4% at 1.0 V. This suggests that starvation makes the problem harder to solve accurately. Either way, the point is the model massively reduces the complexity of computing the 2D transport problem and can enable computations that would otherwise cause the simulation to crash.

Compared to the earlier work on the mD model,^18^ the results here are similar. The experimental results suggest that the lowest concentration is reached at the same time independent of the initial concentration, and the new model captures this part slightly better. As for the experimental data, it is also a bit smoother in the 20–80 min timeframe, which could be linked to the enhanced stability.

#### Adsorption ODE and Optimization

For the next step, providing partial coupling between the adsorption and the spatial information could improve the detailed resolution and predictive value for new operations and systems.

Let us, therefore, focus on the spatially resolved DL model. The advantage of the DL model is that it uses ODEs instead of PDEs like the previous models (eq 12). This means the adsorption rate depends only on the local concentration and adsorption. Thus, the COMSOL module for chemical reactions can be used to smoothly integrate the spatially resolved DL model with a water-flow simulation, which means that the salt adsorption in CDI is effectively treated as a chemical reaction in a porous catalyst. This also allows the COMSOL’s Optimization module to automatically estimate the unknown parameters from the calibration data.

**Figure 7:**
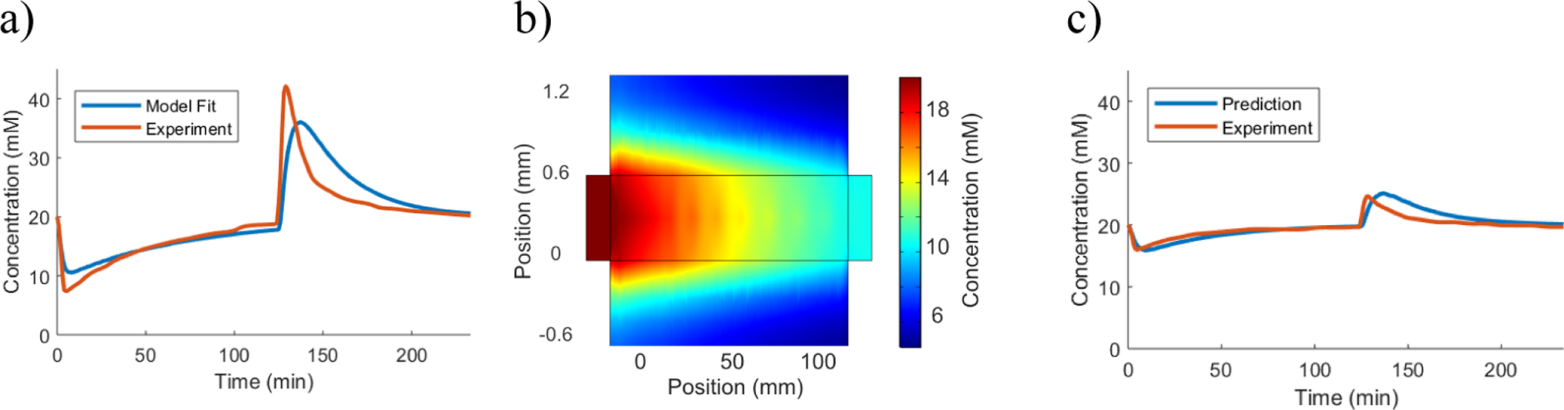
(a) Model fit of the spatially integrated DL model (eq 12) to experimental data from ref (18). (b) Simulated ion-concentration distribution inside the cell at the point of the lowest effluent concentration. (c) Fitted DL model accurately predicts the effluent concentration at 0.4 V for the same device.

An interesting feature in systems with lower ionic concentrations is that the adsorption phase becomes slower,^18^ which can be seen in the experimental data of Figure 7a from the desalination and regeneration phases. Here, previous 0D work in the DL model could not capture this effect^23,70^ but the spatially resolved DL model accurately captures this effect because the ion concentration now locally determines the DL adsorption rate (eq 12, Figure 7). Crucially, this demonstrates that a Langmuir-based approach can be relevant for modeling CDI in 2D and has distinct advantages over comparable methods.

Compared to the previous work on the mD model,^18^ both models have good performance but seem to be a bit too slow at reaching the lowest/highest points. The mD model was symmetrically slower in both the desalination and regeneration phases, while the DL model just underestimated the speed in the regeneration phase. Similar issues with the time scale can also be seen in other works, such as ref (37).

### Scaling Up CDI

Because loose coupling increases computational stability, the two presented methods open up modeling studies of complex CDI architectures. Consider an architecture with two stacked cells of the type investigated previously, with open inlet, outlet, and separator regions (the same as refs (53) and (75)). Due to the complex flows inside the system, such a setup is a good application for a model with relaxed coupling to boost stability.

For the decoupled circuit model, the total 0D adsorption relates to a single-cell calibration because two identical cells adsorb about twice as much as one. This principle readily extends to other variations for which the 0D adsorption can be predicted, such as voltage, ion concentration, and electrode asymmetry. Similarly, changes in flow rates or flow patterns are computed separately.

**Figure 8:**
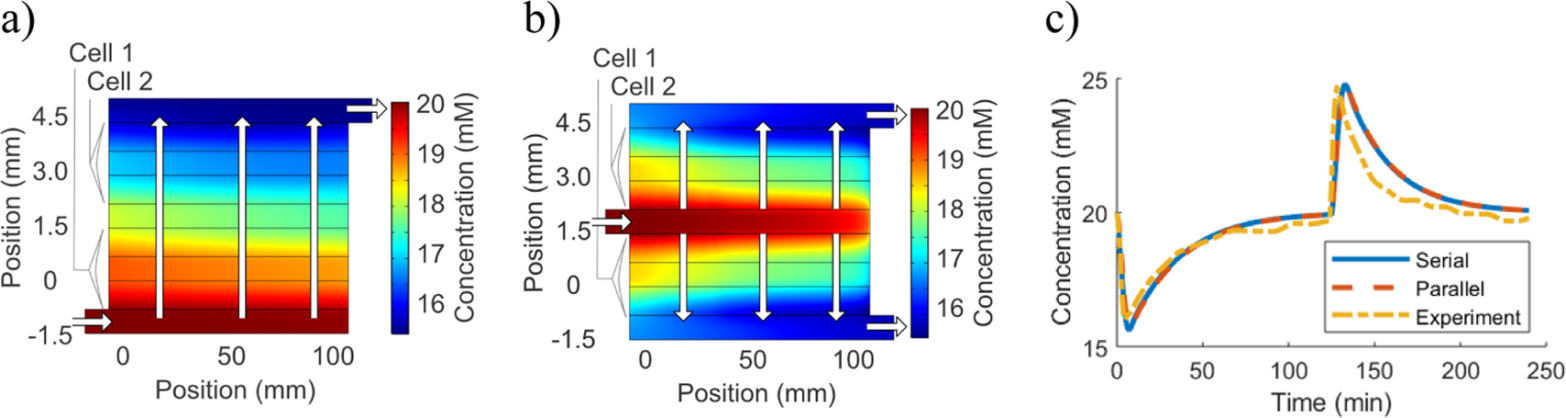
Simulation uses a fitting of the DL model to 0.4 V data from the flow-between device in ref (18), for a single cell. The simulation then evaluates the performance ofo a serial/parallel module with twice the size and flow rate. Note that the arrows show the flow directions, which are through the stacks and uniform across the length of the devices. (a,b) Module’s interior ion concentration at the point of lowest effluent concentration, for a serial and parallel flow, respectively. (c) Effluent concentration for the experimental single-cell module and the simulated two-cell serial/parallel modules with the double-flow rate.

With more spatial information, the fitted DL model from Figure 7c steadily simulates the behavior of the two-cell systems (Figure 8a,b). Notably, the pressure drop in the parallel system is only a quarter of the corresponding serial pressure drop,^53^ while the difference in desalination performance is minuscule (Figure 8c). This means the parallel system requires less pumping pressure for the same output, which makes it energetically superior. Figure 8c also interestingly shows that the global effluent-concentration performance for the single-cell module is practically the same as the corresponding larger system, where the flow rate is doubled as well as the size. Thus, the performance depends mainly on the global replacement rate *Q*/ν_cell_ (*Q* is the flow rate and ν_cell_ is the cell-free volume), which means a 0D model could also be effective for predicting the non-starved performance of larger modular systems.

## Limitations and Prospects

This work has found two approaches to beneficially relax complexities of 2D modeling in CDI, and below follows a discussion on the limitations and prospects of these methods.

One may reflect that the cell’s interior structure is required to realistically calculate ion adsorption. This is partially true because modeling adsorption through 0D calculations should normally miss system aspects related to size, such as diffusion-limited adsorption rates at low ionic concentrations. However, as the Randles series resistance represents all rate-limiting effects, the resistance varies depending on the operating conditions, meaning that the 0D adsorption model accurately fits the adsorption dynamics even at low ionic concentrations. Thus, more accurately, the practical limitation of the 0D model is that it neglects the resistance differences between starved and normal conditions, which could reduce the predictive value over large variations in the inlet concentration. One way of handling this could be to adopt a concentration-dependent resistance formulation.^76^

Another feature that separates the presented approach is that both models assume that the governing equations are the same throughout the cell. However, some specific changes, such as increasing the electrode thickness, may non-linearly alter the total adsorption, which corresponds to a change in the per-volume adsorption and desorption constants. This means researchers implementing this method should consider if, and how, changes in operational and structural conditions affect the system parameters. The current work has explicitly investigated global variations in voltage and flow rate, local variations in ion concentrations, and cell-count upscaling. For future work, literature reports additionally provide ample inspiration for how to address changes in voltage,^23,24^ flow rate,^23^ concentration,^23,24^ electrode size,^24^ electrode surface modifications,^24^ and multi-ion solutions.^30^

## Conclusions

Because interest in the applications of CDI is growing, 2D-modeling of large-scale interconnected CDI devices is becoming increasingly important. The dominant 2D-modeling approaches today employ computationally complex 2D-PDE systems for predicting ion adsorption in small devices.^18,62^ Therefore, we have proposed and validated a relaxed adsorption-flow coupling approach. The relaxation allows simulations to reach the state-of-the-art modeling accuracy with enhanced stability that greatly expands its tractability for realistic large-scale modeling. This work also demonstrated that a Langmuir-based approach can simulate internal and global dynamics of the desalination process with CDI devices.

The present work discovered two routes to achieving relaxed 2D coupling. In the first approach, a 0D method separately calculates the ion adsorption, which is then uniformly integrated into a general flow simulation. Experimental data show that the model generates surprisingly accurate global results and can reliably identify concentration shocks that limit the effective adsorption rate. In the second approach, a localized steady ODE model for adsorption holistically combines with the background water-flow simulation. Thus, the modeling structure firmly relaxes the calculations, which means it reliably extends to large-scale CDI systems with varying concentrations and complex flows.

## Notes

The data and COMSOL model underlying this study are openly available in Mendeley Data at Nordstrand, Johan (2022), “COMSOL Model for: Langmuir-Based Modeling Produces Steady Two-Dimensional Simulations of Capacitive Deionization via Relaxed Adsorption-Flow Coupling”, Mendeley Data, V1, doi: 10.17632/b55r8ybmdp.1.
